# Behavioral and inflammatory sex differences revealed by celecoxib nanotherapeutic treatment of peripheral neuroinflammation

**DOI:** 10.1038/s41598-022-12248-8

**Published:** 2022-05-30

**Authors:** Brooke Deal, Laura M. Reynolds, Charles Patterson, Jelena M. Janjic, John A. Pollock

**Affiliations:** 1 Department of Biological Sciences, Bayer School of Natural and Environmental Sciences, Duquesne University, Pittsburgh, PA USA; 2 Chronic Pain Research Consortium, Duquesne University, Pittsburgh, PA USA; 3 Graduate School of Pharmaceutical Sciences, Duquesne University, Pittsburgh, PA USA; 4 College of Life Sciences, Thomas Jefferson University, Philadelphia, PA USA

**Keywords:** Nanobiotechnology, Drug delivery, Chronic inflammation, Somatic system, Neuroimmunology, Chronic pain, Monocytes and macrophages, Neuropathic pain, Peripheral neuropathies

## Abstract

Neuropathic pain affects millions of people worldwide, yet the molecular mechanisms of how it develops and persists are poorly understood. Given that males have historically been utilized as the primary sex in preclinical studies, less is known about the female neuroinflammatory response to injury, formation of pain, or response to pain-relieving therapies. Macrophages contribute to the development of neuroinflammatory pain via the activation of their cyclooxygenase-2 (COX-2) enzyme, which leads to the production of prostaglandin E_2_ (PGE_2_). PGE_2_ activates nociception and influences additional leukocyte infiltration. Attenuation of COX-2 activity decreases inflammatory pain, most commonly achieved by nonsteroidal anti-inflammatory drugs (NSAIDs), yet NSAIDs are considered ineffective for neuropathic pain due to off target toxicity. Using chronic constriction injury of the rat sciatic nerve, we show that males and females exhibit quantitatively the same degree of mechanical allodynia post injury. Furthermore, a low-dose nanotherapeutic containing the NSAID celecoxib is phagocytosed by circulating monocytes that then naturally accumulate at sites of injury as macrophages. Using this nanotherapeutic, we show that treated males exhibit complete reversal of hypersensitivity, while the same dose of nanotherapeutic in females provides an attenuated relief. The difference in behavioral response to the nanotherapy is reflected in the reduction of infiltrating macrophages at the site of injury. The observations contained in this study reinforce the notion that female neuroinflammation is different than males.

## Introduction

Neuroinflammation is typically caused by injury, infection, or neurodegenerative disorders^1^. A hallmark of neuroinflammation includes rapid infiltration of immune cells at the site of injury that modulate the release of cytokines and chemokines^2^. As a result, specific inflammatory responses, such as cellular and molecular signaling, influence tissue repair, while other components of the inflammatory dialogue cause the sensitization of somatosensory neurons, driving them to hypersensitivity^3^. When these physiological processes are unregulated, the neuroinflammatory response can result in hypersensitivity and neuropathic pain. Furthermore, dysregulated neuroinflammation can interfere with the regenerative potential of the inflammatory response, impeding neuroregeneration and tissue functional recovery following injury^4^.

At a cellular level, neuroinflammation is a complex cross-communication occurring between immune cells, neuronal cells, and glial cells that participates in teetering the cellular milieu between proinflammatory and anti-inflammatory^5–7^. Macrophages play a central role in this mediation, functioning at several levels in the inflammatory response as well as in the repair and regeneration of peripheral neurons. Once stimulated, one of the functions of macrophages is the activation of cyclooxygenase-2 (COX-2), an enzyme that in turn produces prostaglandin E_2_ (PGE_2_). The presence of PGE_2_ has been linked to neuronal sensitization and pain through the increase of intracellular cAMP and Ca^+^, allowing for heightened neurotransmitter release^8–11^. COX-2 and PGE_2_ production has been a target for inhibition by many pharmaceuticals, including aspirin, ibuprofen, and celecoxib^12^. COX-2 inhibitors work by reducing the production of PGE_2_, attenuating inflammation, and thereby providing pain relief. However, a problem with COX-2 inhibitors is that due to the systemic dosage needed to achieve neuropathic pain relief from nerve injury and the associated reduction in inflammation at the site of injury, there are risks of off-target toxicity such as gastrointestinal bleeding, heart attack, stroke, and kidney damage^13^. As such, conventional non-steroidal anti-inflammatory drugs (NSAIDs) and COX-2 inhibitors were shown to be ineffective in the treatment of neuropathic pain^14^.

One of the reasons for the need for high dosages is poor bioavailability of the drugs. In our previous work, we demonstrated that by directing the COX-2 inhibitor celecoxib (CXB) to macrophages by natural phagocytosis of the nanotherapeutic formulation, both reduced systemic exposure and increased overall drug efficacy were achieved^15,16^. In these studies, a single intravenous dose of celecoxib nanotherapeutic (CXB-NE) delivering 0.24 mg/kg celecoxib was found to provide 6 days of statistically significant relief from pain-like behavior caused by surgical chronic constriction injury (CCI) of the male rat sciatic nerve^15,16^. When compared to oral administration, this single dose represents approximately 2000-fold less drug required to alleviate mechanical hypersensitivity for these 6 days^15–18^. This single dose is effective upon intravenous injection because circulating monocytes phagocytose the nanoemulsion and then naturally aggregate at the site of neuronal injury^15^ or inflammatory insult^19^. Instead of an effective systemic dose, which presents the drug to all tissues, the nanotherapeutic functions with macrophages acting as both a target and a vehicle. This allows for the drug to be brought to the site of inflammation, where macrophage-specific COX-2 inhibition attenuates the production of PGE_2_. While recent work has evaluated CXB-NE’s ability to reduce inflammatory signaling in male and female mice experiencing acute inflammation resulting from injection of Freund’s Complete Adjuvant^19^, CXB-NE has not yet been evaluated in female neuroinflammation and neuronal injury models.

In addition to macrophages exerting inflammatory effects at the site of injury, associated spinal microglia are reactive to peripheral inflammation in ways that can enhance pain signaling in the central nervous system^20^. Intrathecal injection of drugs can disrupt the functional proinflammatory role that activated microglia play in nociceptive signaling, preventing pain-like behavior in rodents^21^. However, blocking microglial cells alleviates pain in only males and not female rodents^22^. Interestingly, in mice that lack T cells, blocking microglia leads to pain relief in both sexes^23,24^, and when T cells are restored, pain-like behavior returns only in female mice. This points to an influential role of T cells in the development of female neuroinflammatory pain. This is particularly interesting because during a typical neuroinflammatory response, macrophages and T cells directly interact with one another through their major histocompatibility complex (MHC-II) and T cell receptor (TCR), respectively. Macrophages infiltrate the site of injury within hours to days of injury. These infiltrating macrophages release cytokines and chemokines that attract T cells in the following days^25^. Once T cells communicate with antigen-presenting cells such as macrophages, they too contribute to the proinflammatory or anti-inflammatory milieu. Other studies have seen that without the infiltration of phagocytic cells such as macrophages, T cells are unable to infiltrate the nerve^26^. These observations reinforce the need to assess the influence of CXB-NE on macrophages and their relationship to the role of T cells in the neuroinflammatory response in both males and females.

In this study, we examined sex-specific relief from neuroinflammation and hypersensitivity following CCI injury achieved by COX-2 inhibition from a single intravenous injection of CXB-NE. This is of particular importance, because NSAIDs have been previously thought to be ineffective for neuroinflammatory pain relief at safe dosages^14^. We found that male and female rats experiencing CCI of the sciatic nerve exhibited quantitatively equivalent mechanical hypersensitivity as measured by the von Frey technique. When male and female CCI rats were given the same intravenous injection of CXB-NE, male rats exhibited a complete reversal of their mechanical allodynia persisting for five days, whereas female rats achieved only partial relief over the same time course. We also examined the presence of immunological cells within the fasciculated sciatic nerve where the cells can have a direct effect on the nerves. Cells such as CD68-positive macrophages, CD3e-positive T cells, CD4-positive cells, and CD8-positive cells are assessed to explore the sex differences between their involvement in inflammation of the nerve, and the effect of treatment on their presence. Even though males and females exhibit quantitatively the same level of hypersensitivity to nerve injury as well as both having increased macrophages following CCI, the female response to the nanotherapeutic is quantitatively and qualitatively different, revealing previously unknown pharmacodynamic differences between male and female COX-2 inhibition in neuroinflammation.

## Results

### Treatment effect on mechanical allodynia

**Figure 1:**
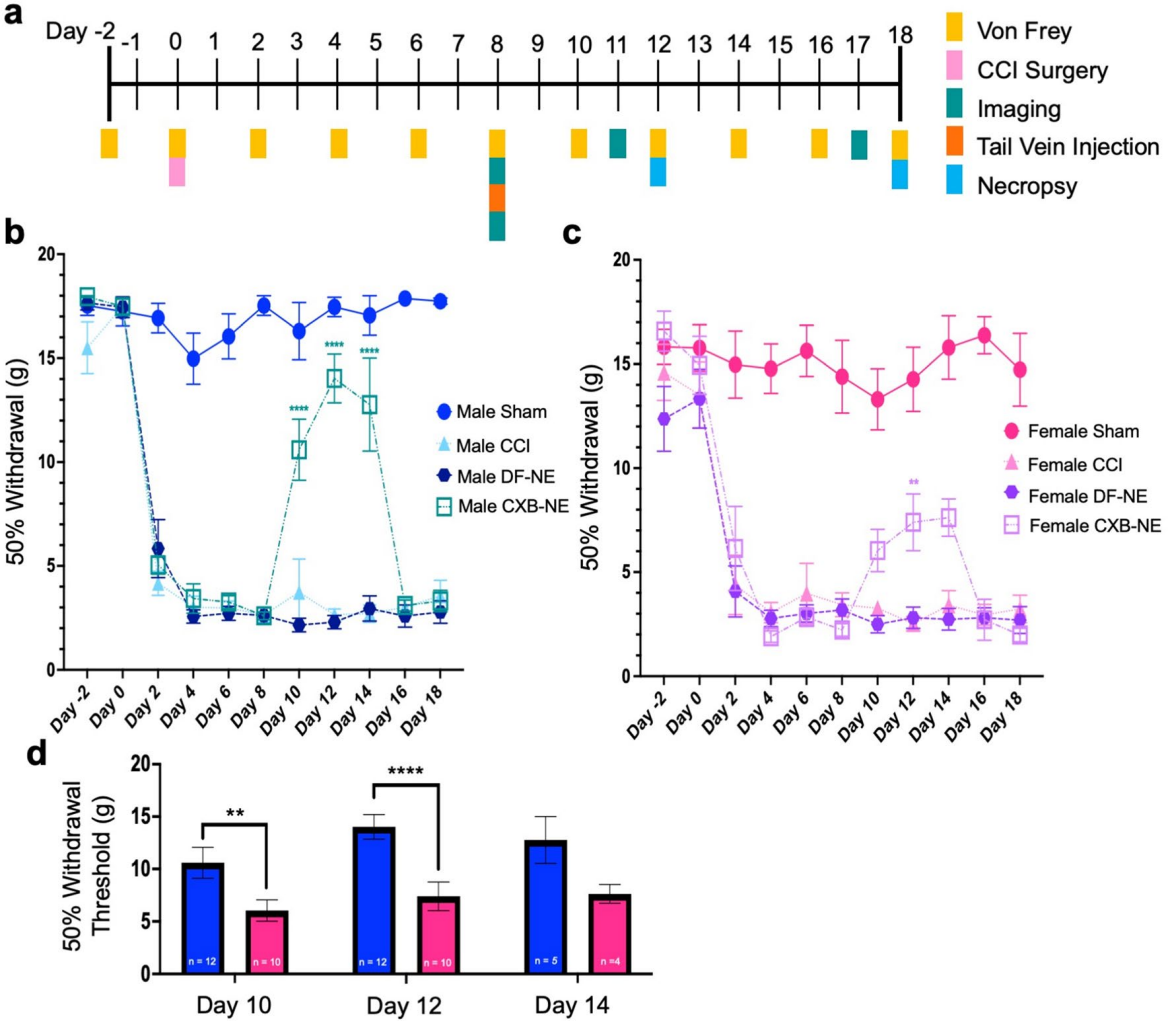
Mechanical hypersensitivity caused by CCI and the difference in pain relief from celecoxib-loaded nanoemulsion (CXB-NE). (**a**) Timeline of animal behavior and drug administration for both male and female groupings. (**b**) Male hyperalgesia and allodynia was significantly relieved for at least 5 days following CXB-NE administration compared to both the CCI and DF-NE treatment groups. (**c**) Female allodynia and hyperalgesia were significantly relieved for 1 day (day 12) after CXB-NE administration via tail vein injection compared to the CCI and DF-NE treatment groups. (**d**) Summary of the sex differences in pain relief in drug-treated CCI animals at days 10, 12, and 14, with females (pink) experiencing significantly less relief than males (blue). Data is displayed as averages ± SEM. *≤ 0.05, **≤ 0.01, ***≤ 0.001, ****≤ 0.0001.

Mechanical sensitivity was measured every other day prior to and after CCI surgery on the sciatic nerve using the up-down von Frey method^15,16,27,28^ (Fig. 1a). Once CCI was performed, male and female rats exhibited quantitatively equivalent withdrawal thresholds across the experimental time course, ultimately exhibiting hypersensitivity averaging approximately 2–3 g, which dropped from a baseline of ~ 16 g (Fig. 1b,c). The injection of drug-free nanoemulsion (DF-NE) on day 8 did not exacerbate or alleviate mechanical hypersensitivity in either male or female rats (Fig. 1b,c). When CXB-NE was administered to male rats, significant mechanical hypersensitivity was relieved for 5 days post administration (Fig. 1b) (*Treatment x Time* F_77, 759_ = 8.906, *p* < 0.0001; day 10 *p* ≤ 0.0001, day 12 *p* ≤ 0.0001, day 14 *p* ≤ 0.0001). A similar bell curve response, representative of relief from hypersensitivity, could be seen in females beginning with the first measurement taken after injection on day 10 and lasting until day 16. However, the female response to CXB-NE was attenuated when compared to males, reaching statistically significant relief for 1 day at day 12 (Fig. 1c) (*Treatment x Time* F_77, 759_ = 8.906, *p* < 0.0001; CXB-NE vs CCI *p* = 0.0031, CXB-NE vs DF-NE *p* = 0.0073). At days 10 (*p* = 0.0061) and 12 postsurgery (*p* < 0.0001), this relief was significantly different between the male and female CXB-NE groups (Fig. 1d).

### Assessment of cellular involvement of neuroinflammation

**Figure 2:**
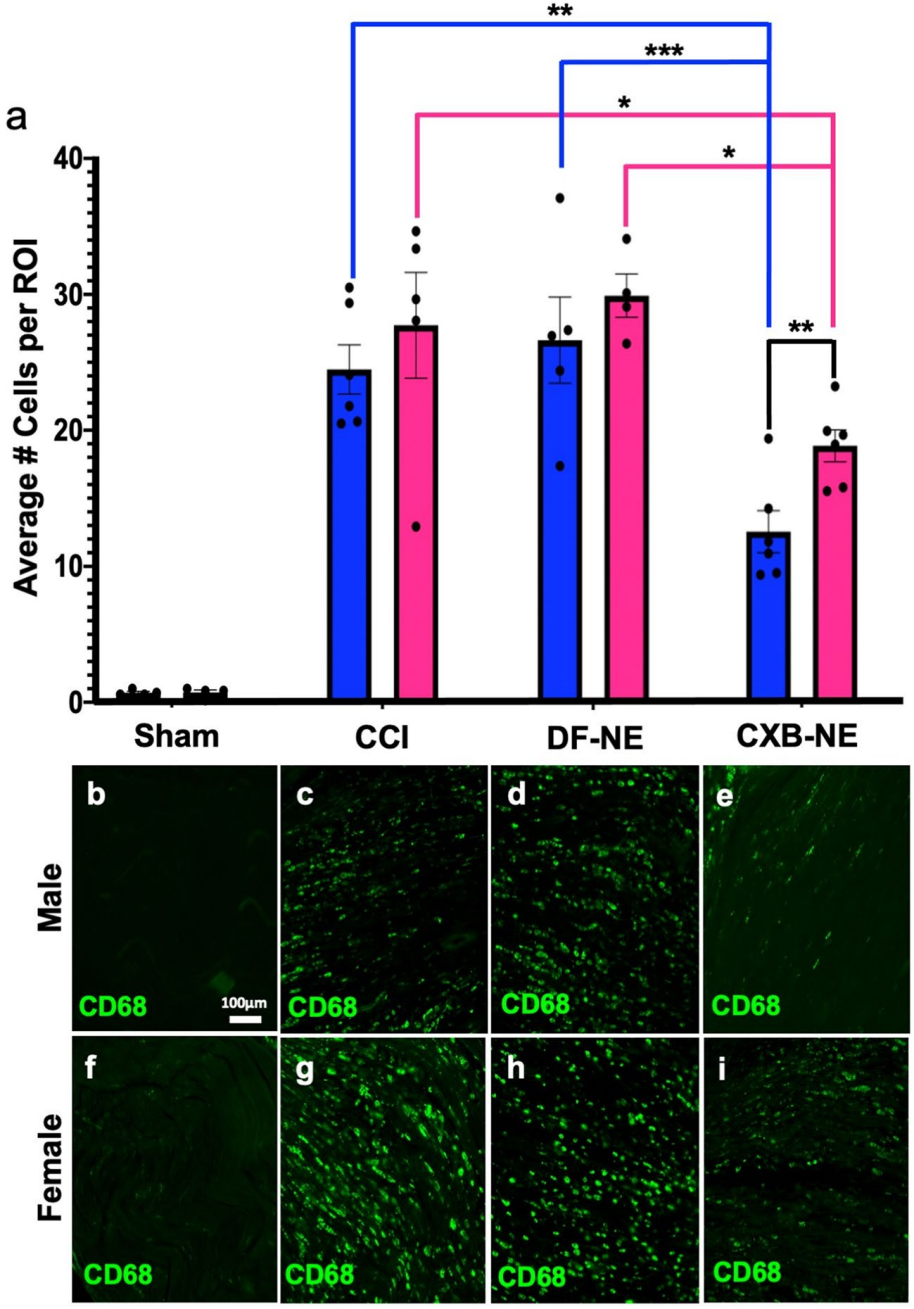
Day 12 macrophage infiltration after CCI decreases significantly in both male and female injured nerves treated with CXB-NE. However, CXB-NE treated females exhibited significantly more infiltrating CD68+ cells than similarly treated males. (**a**) Average number of CD68+ macrophages per region of interest (ROI) across sex (male-blue, female-pink) and condition. Data is displayed as averages ± SEM. *≤ 0.05, **≤ 0.01, ***≤ 0.001, ****≤ 0.0001. (**b**–**i**) Immunofluorescence staining for CD68+ macrophages in the sciatic nerve.

Both males and females treated with CXB-NE experienced the peak of their relief from mechanical hypersensitivity four days following injection (Fig. 1b,c); therefore, a subset of animals were sacrificed on day 12 to examine the changes in the infiltration of immune cells at the site of injury on that day. A second set of animals of both sexes were sacrificed on day 18 to examine cellular infiltration once relief from hypersensitivity had dissipated. The status of the estrous cycle in the females did not affect the results as all the females appeared to be on the same cycle (Supplemental Fig. 1). Furthermore, given that female rats have a 4-day estrous cycle, and that the female progression to and degree of hypersensitivity perfectly matches that of the males (Fig. 1b,c), it appears that the estrous cycle is not playing a significant role in influencing the rat’s behavior for these conditions. Because CXB-NE is phagocytosed and delivered by infiltrating macrophages, the presence of the infiltrating macrophages, which would have the most direct effect on the nerves was quantified in the sciatic nerve (Fig. 2 and Supplementary Fig. 2).

**Figure 3:**
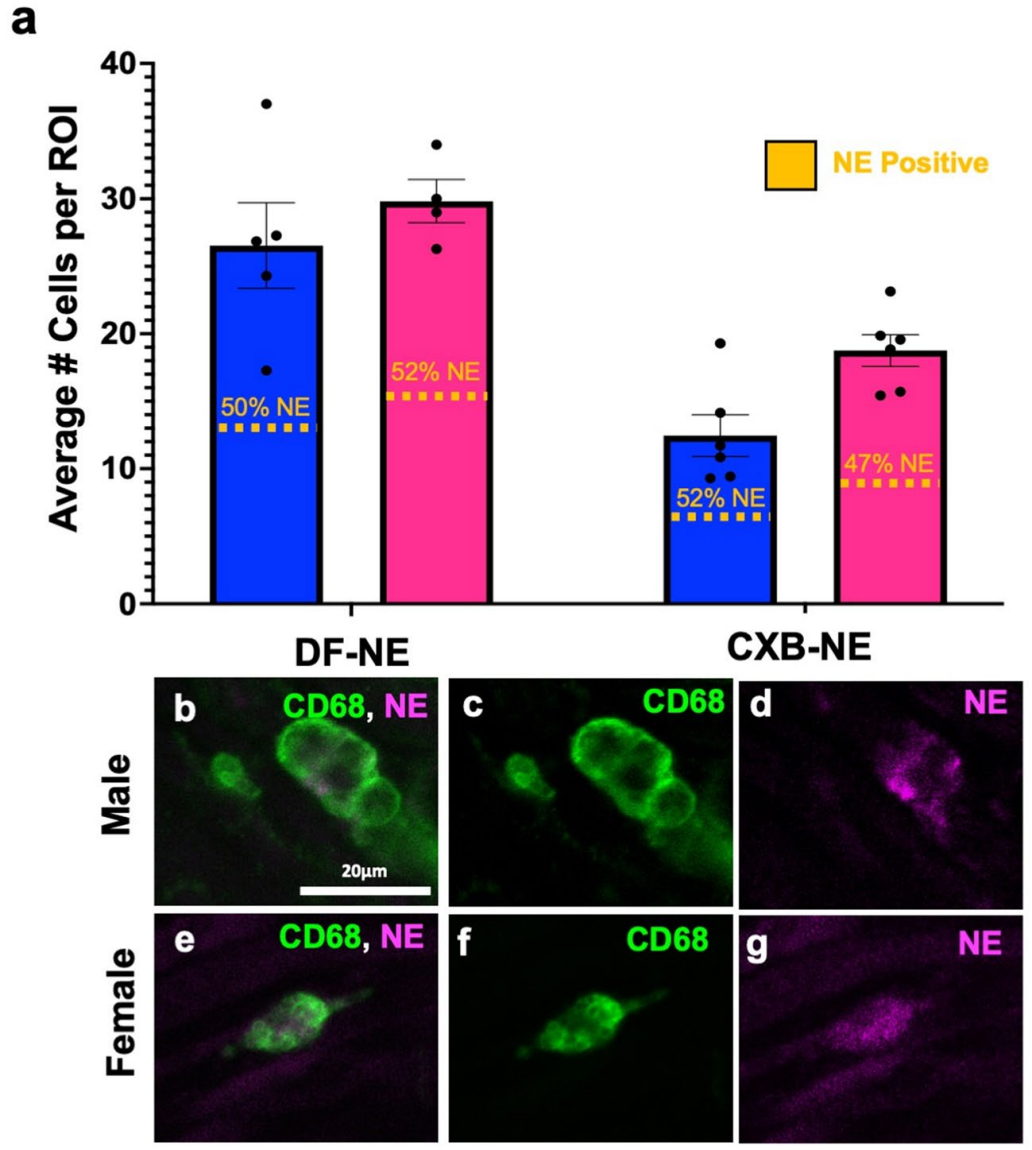
Nanoemulsion is equally phagocytosed by both male and female macrophages. (**a**) Nanoemulsion is taken up by 47–52% of macrophages regardless of sex or treatment. DF-NE n = 5 (male), 4 (female); and CXB-NE n = 6 (male), 6 (female). (**b**–**d**) CD68+ macrophage in male sciatic exhibit internalized nanoemulsion DiR signal (**d**), as do infiltrating macrophages in the injured female sciatic nerve (**e**–**g**) with detectable DiR nanoemulsion (**g**).

During maximum relief at day 12, the sham surgical control had few if any CD68-positive (CD68+) macrophages detected within the sciatic nerve in either sex (Fig. 2b,f). Once CCI was performed, a significant number of infiltrating macrophages could be detected in the sciatic nerve in both males and females (Fig. 2c,g). CCI animals that received an injection of drug free nanoemulsion (DF-NE) exhibited a level of CD68+ macrophages (Fig. 2d,h) that was similar to that seen in CCI only animals (Fig. 2c,g). The animals that were given CXB-NE had a significant reduction in the number of infiltrating macrophages at the site of injury (female F_3,15_ = 28.99, *p* < 0.0001; male F_3,17_ = 30.10, *p* < 0.0001) when compared to their CCI (female *p* = 0.0450, male *p* = 0.0019) and DF-NE (female *p* = 0.0175, male *p* = 0.0006) counterparts (Fig. 2e,i). While day 12 CCI females treated with CXB-NE presented with a significant reduction in the number of infiltrating macrophages compared to their CCI and DF-NE same sex counterparts, they interestingly had significantly more macrophages than males treated with CXB-NE (F_5,5_ = 1.732, *p* = 0.0088). Assessment of the near infrared DiR signal from the nanoemulsion revealed no significant difference between the percentage of macrophages that phagocytosed the nanoemulsion, as observed between the sexes (Fig. 3).

Once male and female CXB-NE rats returned to mechanical hypersensitivity by day 18, the level of macrophage infiltration was equivalent to that of DF-NE treated animals as well as CCI animals that received no treatment (Supplementary Fig. 2).

**Figure 4:**
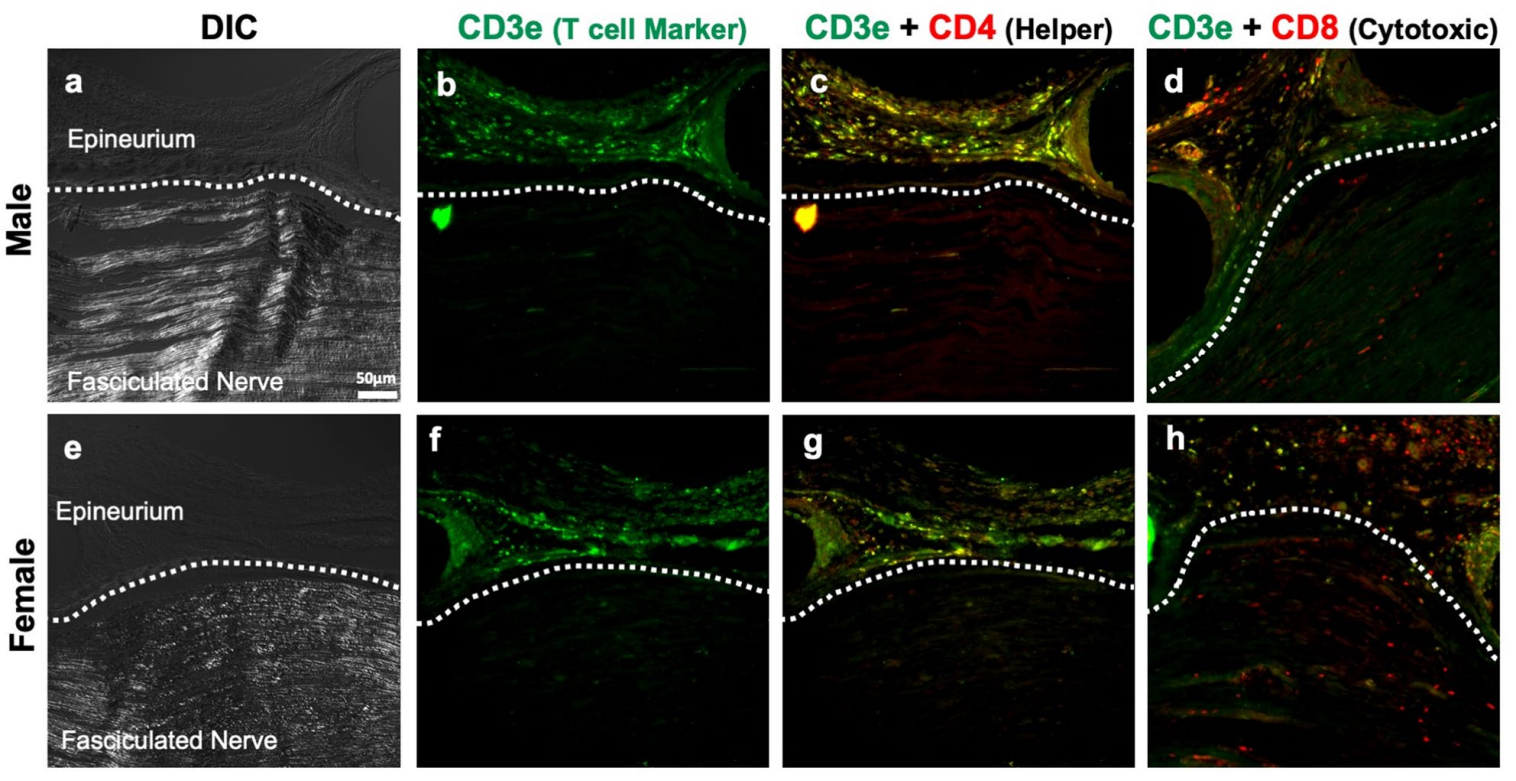
T cells localize to the epineurium of the sciatic nerve, where their cytokines can influence the neuronal cells and Schwann cells. (**a**/**e**) Demonstrate the ability to differentiate the fasciculated nerve from the epineurium using differential interference contrast (DIC). (**b**/**f**) The CD3e pan-T cell marker shows localization of T cells in the epineurium. (**c**/**g**) Shows that the CD4 subtype marker (red) co-localizes with CD3e (green) heavily in the epineurium with sparse occasional CD4 only staining occurring within the fasciculated nerve. (**d**/**h**) Shows that CD8 (red) occasionally co-localizes with CD3e in the epineurium but also is more frequently present without CD3e within the fasciculated nerve. Panels d and h are located from a different slide than panels (**a**–**c**) and (**e**–**g**) due to compatibility of primary antibodies.

**Figure 5:**
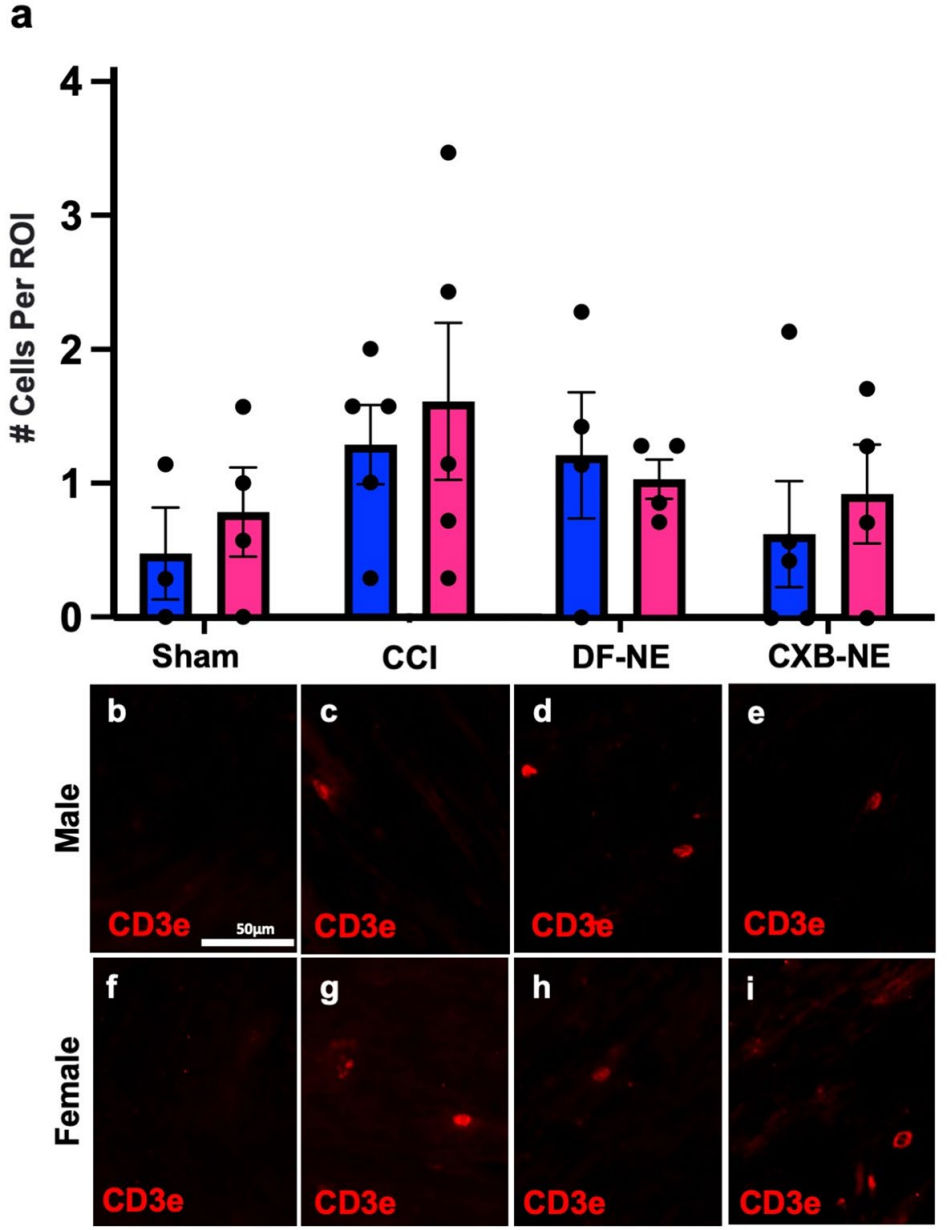
CD3e+ T cells are present at a low density in the fasciculated nerve; their number unchanged by CCI surgery, DF-NE or CXB-NE drug delivery. Data is displayed as averages ± SEM. Sham n = 3 (male), 4 (female); CCI n = 5 (male), 5 (female); DF-NE n = 4 (male), 4 (female); and CXB-NE n = 5 (male), 4 (female).

**Figure 6:**
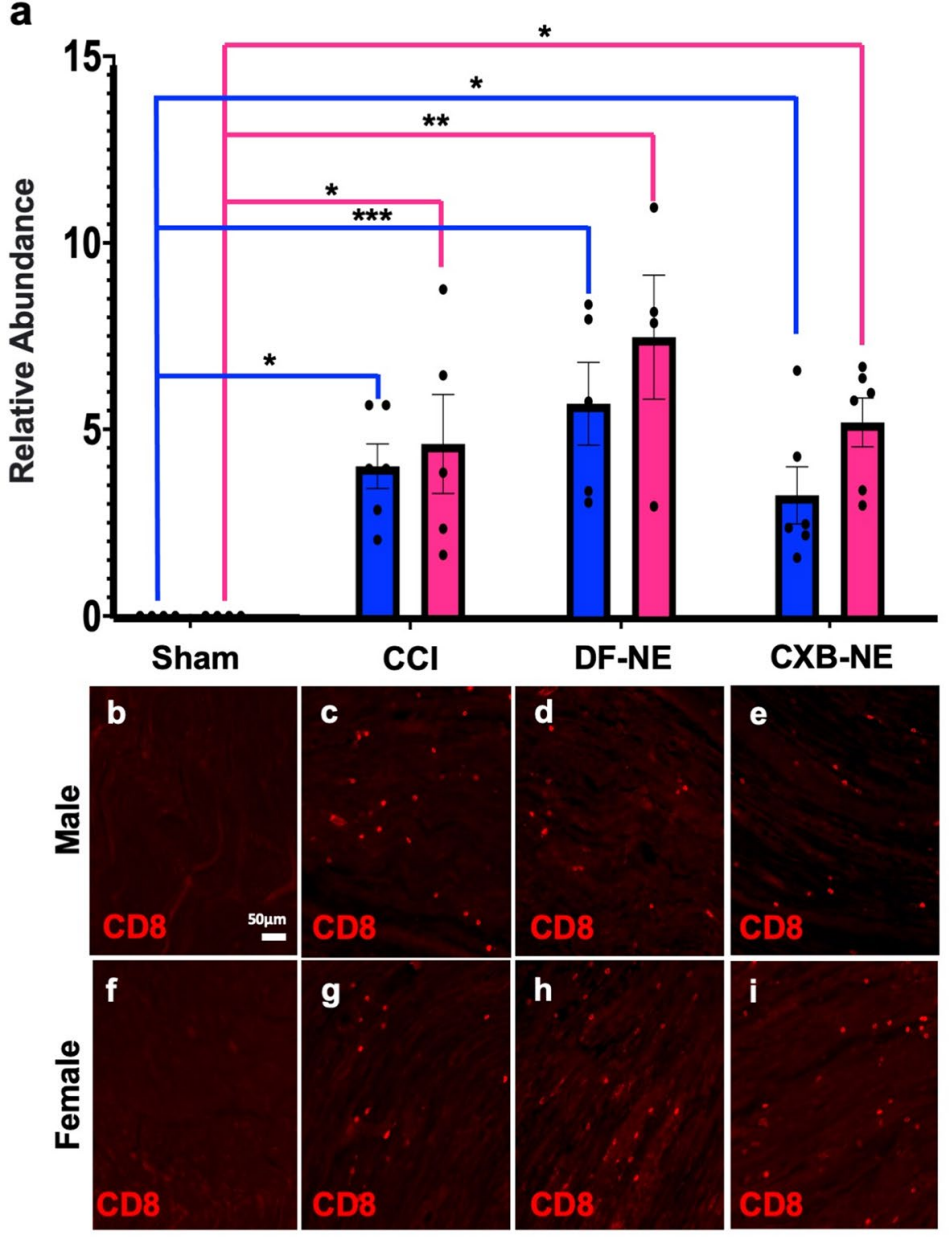
CD8+ cells are recruited to the site of injury after CCI and are not significantly affected by CXB-NE treatment but exhibit the trend to have higher infiltration in females than males. (**a**) CD8+ cell recruitment into the injured nerve by sex (female-pink, male-blue). Data is displayed as averages ± SEM. *≤ 0.05, **≤ 0.01, ***≤ 0.001, ****≤ 0.0001. (**b**–**i**) Immunofluorescence staining for CD8+ cells in the sciatic nerve.

In order to examine the influence of the decreased number of inflammatory macrophages on other immune cells resulting from CXB-NE treatment, we examined the cell-specific expression of CD3e, CD4 and CD8 by immunohistochemistry. When the sciatic nerve was examined 12 days post CCI, the majority of the CD3e+ T cells were localized to the epineurium (Fig. 4b,f), and little to no T cells were found within the nerve bundle itself in either sex or any condition (Fig. 5). While examining CD3e positive T cells, tissues were also co-stained for CD8 to visualize cytotoxic T cells that were positive for both CD3e and CD8^26^ (Fig. 4d,h), as well as CD4 to visualize helper T cells that were positive for both CD3e and CD4^29^ (Fig. 4c,g). Even though the vast majority of T cells were localized in the epineurium of both males and females (Fig. 4b,f), cells singularly stained with CD8 could be seen infiltrating the fasciculated nervous tissue (Fig. 4d,h). We chose to quantify these CD8+ immune cells in the nerve track. Animals that underwent sham surgery had no CD8+ cell infiltration (Fig. 6b,f). All animals who underwent CCI surgery with or without injection of nanoemulsion had a significant increase in CD8+ cells (Fig. 6c–e,g–i) compared to the sham surgical control. Although there was no significant effect of CXB-NE treatment on CD8+ cells in either males or females, the female CCI, DF-NE, and CXB-NE groups all tended to have a higher relative abundance of CD8+ cells at the site of injury than their male counterparts.

## Discussion

Here, we demonstrate that under the same surgical conditions of nerve injury (CCI), male and female rats exhibit transitions to quantitatively equivalent hypersensitivity. Furthermore, we demonstrate for the first time that in females, a single low-dose injection of CXB-NE can reduce neuroinflammation and mechanical hypersensitivity. However, this reduction is not as effective as in male rats similarly treated^15,16,30,31^. We are therefore able to investigate how female neuroinflammation and associated hypersensitivity can be attenuated using celecoxib nanotherapy. This is noteworthy because previously, the use of COX-2 inhibitors were not thought to be effective in treating female neuroinflammation at non-toxic dosages^14^. The histological observations reveal that the density of infiltrating macrophages at the site of neuronal injury correlates with behavioral hypersensitivity. CCI females that were treated with CXB-NE exhibited a significant reduction in the number of infiltrating macrophages in the injured nerve as compared to female controls and behaviorally, they exhibited attenuated hypersensitivity. However, females did not achieve the same degree of relief from hypersensitivity as that observed in males similarly treated. Correspondingly, while the CXB-NE treated females had fewer infiltrating macrophages at the site of injury than the females experiencing hypersensitivity, they nonetheless exhibited a higher degree of inflammation than males. Assessment of the phagocytosis of the nanotherapeutic revealed that there was equivalent engulfment of nanotherapy by macrophages between sexes. Thus, these observations led us to investigate whether CXB-NE would alter the infiltration of other immune cells into the site of injury, such as T cells, which have been shown to be sexually dimorphic at influencing inflammatory pain^23,24^. Taken together, the use of CXB-NE COX-2 inhibiting nanotherapy reveals a pharmacodynamic sex difference in neuroinflammation and relief from hypersensitivity.

In order to examine sex differences in mechanical hypersensitivity relief, both male and female rats underwent CCI of the sciatic nerve. Mechanical withdrawal was measured before and after treatment with the nanotherapeutic. Furthermore, to examine the involvement of inflammatory cells, the affected sciatic nerve tissues were collected at the peak of pain relief as well as at day 18 once hypersensitivity had returned. Here, we show that male and female rats that have experienced identical CCI of the right sciatic nerve exhibit an equivalent progression to mechanical hypersensitivity. In other words, males and females feel the same amount of pain with the same nerve injury. This study also found that male and female rats were both significantly relieved with a single injection of CXB-NE (Fig. 1b,c). However, when these injured male and female rats were treated with a nanotherapeutic containing the NSAID celecoxib, we showed that while females achieved significant pain relief, they did not achieve the same level of pain relief as males (Fig. 1d). This sex difference in pain relief correlates with the density of infiltrating macrophages in the CXB-NE condition (Fig. 2). A few studies of NSAIDS in both sexes have also seen unequal relief from NSAID usage in females when compared to males. In one of these studies, healthy volunteers experienced induced inflammatory pain; the authors then assessed the ability of the nonselective COX inhibitor ibuprofen to provide relief. Here they found that male volunteers but not females received analgesia^32^. Chillingworth and colleagues^33^ extended on these differences by examining COX involvement in hypersensitivity in mice. They showed that in the inflammatory CFA model, COX-1 and COX-2 appear to play an additive role in female inflammation formation, while in males, the pain response is mainly driven by COX-2. Another study, exploring male and female response to CFA treatment in rats, found a similar phenomenon to that of Chillingworth et al^33^ with the nonselective COX inhibitors working the best in females to reduce allodynia and the COX-2 inhibitor working the best in males to reduce inflammation^34^. Our study further adds to these findings by being the first to evaluate sex differences by inhibition of COX-2 in neuroinflammation with a celecoxib nanoemulsion (CXB-NE). Here we revealed sex differences resulting from the inhibition of COX-2 in both mechanical hypersensitivity and the extent of macrophage-driven inflammation. The results presented here, along with those of Chillingworth^33^ and Craft^34^, indicate that inhibition of COX-2 is most effective in providing relief for male neuroinflammation, while relief in females appear to include involvement of COX-2 activation as well as other factors that contribute to their neuroinflammatory hypersensitivity.

While both males and females experience activation and recruitment of infiltrating macrophages during a neuroinflammatory response of an injured nerve, females have heightened responses to PGE_2_ postinjury^35^. This fact along with the fact that there are more infiltrating macrophages could contribute to the behavioral difference in the females treated with CXB-NE where the relief from hypersensitivity is less than that of males receiving an equivalent dose. Why there are more macrophages infiltrating the injured nerve in females and what other underlying fundamental biological differences exist between males and females is unclear. Nonetheless, this is an important finding given that COX inhibitors are one of the most commonly prescribed pain treatments. Although NSAIDs have been shown to be effective in reducing inflammatory pain in both sexes^36^, the research on the sex-specific efficacy of NSAIDs for neuroinflammatory pain has been limited. One of the challenges is that with limited bioavailability of NSAIDs, safe systemic dosages are insufficient for providing effective therapy for human neuropathic pain^14^. Despite this fact, the composition of CXB-NE and the nanoemulsion’s high surface-to-volume ratio increases the bioavailability of celecoxib^37^. Coupled with the fact that the nanotherapeutic is internalized in the cytoplasm of macrophages, the cells that celecoxib can act on, the effective dosage is dramatically less than what is needed compared to oral systemic therapy^15,16^. In this way, the pharmacology of CXB-NE allows it to be effective in treating the hypersensitivity that is associated with neuroinflammatory pain. The increased efficacy at a low dosage avoids toxicity to off-target tissues and organs and is achieved by cell-specific delivery of the drug precisely in the injured tissue. To our knowledge, this is the first time that a safe systemic dosage of COX-2 inhibition has been reported to be effective at relieving female neuroinflammatory pain.

We also explored inflammation at the cellular level during CCI neuronal injury, demonstrating that macrophages infiltrate the injured nerve (Fig. 2). During maximum pain relief from CXB-NE, macrophages are still present at the site of injury; however, there is a significant decrease in their numbers when compared to both male and female DF-NE and CCI conditions. Here, we reveal that the response to CXB-NE differs between males and females at both the behavioral and cellular levels (Figs. 1 and 2). Furthermore, we show that when hypersensitivity returns, CXB-NE treated sciatic nerves exhibit to a similar macrophage infiltration as that of the DF-NE and CCI conditions (Supplementary Fig. 2). This suggests that the presence of macrophages within the nerve directly correlates to the mechanical hypersensitivity being experienced. Furthermore, the difference in relief seen by males and females is not due to the percentage of infiltrating macrophages carrying CXB-NE or DF-NE as equivalent nanoemulsion uptake was observed by both sexes (Fig. 3). Thus, it is not the ability to phagocytose the nanoemulsion and, in turn, deliver the nanotherapy to the site of injury that is causing the difference in the relief from mechanical hyperalgesia or the level of inflammation, but how the attenuation of COX-2 causes differing neuroimmunological communication within the tissue for each of the sexes.

These data appear to contradict what others have reported about sex differences in phagocytic activity^38,39^. However, this prior observation involved an extremely different experimental model. A primary culture of splenic macrophages was derived from gonadectomized lizards, the wild gecko (*Hemidactylus flaviviridis*), which may function differently than macrophages in live rats. Mondal and Rai^38^ treated these gecko-derived macrophages with dihydrotestosterone (DHT) or 17beta-estradiol (E2) at varying concentrations and found differences in phagocytic activity where the lizard cell culture macrophages treated with E2 had a higher rate of phagocytosis than those treated with DHT^38^.

The transition to chronic pain is known to involve the interaction of the nervous system and the immune system^40^. Given that other immune cells could be affected by the attenuation of macrophage COX-2 activity with CXB-NE^16,19^ we focused specifically on T cells because they appear to have a unique contribution to female neuroinflammatory pain^39,41,42^. Furthermore, T cells can interact directly with macrophages during a neuroinflammatory response. Antigen-presenting cells such as macrophages can trigger a pro- or anti-inflammatory phenotype of T cells by affecting the cellular environment^43^. PGE_2_ is able to alter regulatory T cells to inhibit activation, thereby attenuating neuroinflammation^44^. Helper T cells (CD3e and CD4 positive) have also been shown to help decrease neuroinflammation through the use of IL-10, while the role of cytotoxic T cells (CD3e and CD8 positive) is not well known^26^. While there was no difference between sex or treatment for the presence of T cells in the fasciculated nerve (Fig. 5), we did find that both cytotoxic and regulatory T cells were localized within the epineural sheath of the sciatic nerve near the site of injury rather than within the fasciculated nerve bundle (Fig. 4). Considering that these T cells appear to be confined to the epineural sheath, they nonetheless may influence neuropathy from afar via cytokine signaling rather than direct communication with the neurons themselves. IL-10 is a major cytokine used to communicate between macrophages and T cells influencing an anti-inflammatory response as well as aid in neuroregeneration^45^. Previous work has shown that IL-10 is essentially shut down shortly after CCI^46^. These findings regarding T cells are of particular interest because the majority of studies stating that T cells influence sexual dimorphism in the inflammatory response used indirect methods (flow cytometry, animal knockout models, adoptive splenocyte transfers, and qPCR^22–24,47,48^) and do not differentiate between the anatomical domains of the injured nerve.

We also observe a trend where a higher number of inflammatory cells appear to infiltrate the site of injury of the female sciatic nerve as compared to the similarly injured male sciatic nerve. The CCI, DF-NE, and CXB-NE conditions all exhibited higher infiltration of CD68+ and CD8+ cells in females (Figs. 2, 6). This observation is consistent with what has been reported by Klein and Flanagan^39^, where females show stronger innate and adaptive immune responses than males. Given that human females exhibit more autoimmune diseases^49^ and stronger reactions to immunization^50^, it is of interest to consider that females may also have a sex specific peripheral neuroinflammatory response. Therefore, it is important that the evaluation of therapeutics is achieved in both sexes, as the immune responses to disease and treatment differ.

## Conclusion

Here, we demonstrated that equivalent nerve injury results in quantitatively equivalent hypersensitivity in males and females. Our novel finding that the celecoxib nanoemulsion, CXB-NE, which we previously demonstrated as highly effective in males^15,16,31,51^, is effective in females, but with reduced potency. Given that COX inhibitors are one of the most commonly prescribed analgesics^34^, these sex differences in the response to therapy for neuroinflammation reiterates the need for therapeutics to be examined equally in males and females. Assuming that a given therapeutic will work equivalently in both sexes creates an opportunity for incomplete understanding of its effectiveness or its underlying fundamental biology^40^. Given the call to action to find a novel, non-opioid analgesic^40^, the findings shown here are therefore, of great importance. Overall, these results show that there are similarities in the inception and perception of neuroinflammatory hypersensitivity between sexes, where both males and females report the same degree of pain-like behavior from equivalent nerve injury. Despite this, identical treatment with celecoxib nanotherapy reveals a sex difference in the level of relief from hypersensitivity pain-like behavior achieved in chronic constriction injury. These results correlate with the sex difference in the degree of macrophage infiltration in the presence of CXB-NE, as well as the increased number of CD8+ cells at the site of injury in females. Lastly, our deeper evaluation of T cell involvement in neuroinflammation led to the discovery of T cells being confined to the epineural sheath and that the cytokines that they express may influence the injured nerves from that adjacent location. Our findings, along with others^19,33–35^, further emphasize the need to clarify the mechanistic differences in the action of COX inhibition on the inflammatory milieu that result in these sex-specific differences in inflammation and in relief from hypersensitivity and pain-like behavior.

## Material and methods

### Ethics statement

The study was approved by the Ethics committee/Institutional Review Board of Duquesne University. Male (200–250 g) and female (150–200 g) Sprague–Dawley rats were purchased from Hilltop Lab Animals, Inc. (Springdale, PA). These experiments were carried out following the recommendations in the Guide for Care and Use of Laboratory Animals of the National Institutes of Health and the regulations of the Institutional Animal Care and Use Committee (IACUC) at Duquesne University under approved Protocol #1803-02 and in compliance with the ARRIVE guidelines (https://arriveguidelines.org). Rats were acclimated and maintained on a 12:12 h light–dark cycle and housed on paper bedding with access to food and water ad libitum. Starting two days prior to surgery, animals were switched onto a special diet (Research Diets, Inc., New Brunswick NJ; Cat # AIN-93G) to avoid autofluorescence during near infrared imaging of the nanoemulsion in live animals. Animals were socially housed during acclimation. They were then individually housed once von Frey behavior began (two days prior to surgery) to establish baseline behavior; this was done to avoid rats from opening one another’s wounds following surgery.

### CCI surgery

Male and female rats underwent chronic constriction injury (CCI) of the sciatic nerve as described by Bennett and Xie^27^ and in accordance with our prior technique^15,16^ to induce neuropathic pain and inflammation. Four sutures (McKesson 4-0 Chromic Gut Sutures, Cat # S635GX) were loosely tied around the sciatic nerve approximately 1 mm apart in CCI, DF-NE, and CXB-NE treated animals. Sham animals underwent identical surgery to expose the sciatic nerve, but no sutures were tied around the nerve.

### Estrus cycle tracking

Vaginal swabs were collected for 4 consecutive days in order to establish their estrus cycle. Briefly, the vaginal canals of anesthetized rats were flushed with sterile saline and collected on a slide. The slide was allowed to dry. Then cells were fixed with 100% methanol and stained with 5% Giemsa stain for 20 min. Slides were then rinsed in diH_2_O, dried, mounted with Permount (FisherScientific, Cat # SP15-100), and then imaged.

### Behavioral testing

To assess mechanical allodynia and the effectiveness of CXB-NE, von Frey Up-Down analysis was performed as previously described^15,16,28,52^. Testing was performed every other day including baseline behavior from 2 days before and on the day of surgery. Rodents were tested in separate same sex cohorts. Animals were acclimated for 30 min in individual plexiglass chambers with a mesh metal floor to allow access to paws. After acclimation, the left and right hind paws were tested using calibrated von Frey filaments (Stoelting Company, Cat # 58011). Rodents were probed with the filament in the middle of their hind paw for 3 s with the filament bent to a 30° angle. Positive responses were recorded through the visualization of a flick or quick lift. Animals were given a minimum of 30 s between probing of the same paw twice. Animals were randomly assigned to their groupings, and the behavioralist was blinded to animal treatment. Behavioral testing was scheduled as outlined in Fig. 1a.

### COX-2 inhibiting nanotherapeutic

The nanotherapeutic used in this study was produced by microfluidization as reported earlier^15,19^. Briefly, celecoxib was dissolved in hydrocarbon oil, mixed with perfluoro-15-crown-5 ether and nonionic surfactants, and emulsified using high shear processing, microfluidization on a M110 microfluidizer (Microfluidics, Westwood, MA, USA). CXB-NE and DF-NE were formulated with the near-infrared (NIR) fluorescent dye DiR and DiIC18(7) (1,1′-dioctadecyl-3,3,3′,3′ tetramethylindotricarbocyanine iodide) was added to hydrocarbon oil for fluorescent detection in tissues. Nanoemulsions were sterile filtered prior to use in animals and tested for colloidal properties, cell viability and stability as reported earlier^15,19^.

### Tail vein injection

Intravenous injections of DF-NE and CXB-NE were performed 8 days after surgery and following von Frey behavior analyses. Injections and near-infrared imaging inspection of injection quality were performed as described by Saleem et al.^51^. Investigators were blinded to the nanoemulsion treatment. Animals were anesthetized with respiratory isoflurane. Tails were warmed in water for 1 min to dilate the lateral veins. Tails were then dried and sterilized with an alcohol pad. A 27G needle, bevel up, was placed distally into the lateral tail vein. Upon blood flashback, a syringe containing 300μL of DF-NE or CXB-NE (55.5 micrograms of celecoxib) was attached and slowly injected into the vein^15,16,51^; this dose was used for all animals. The quality of the tail vein injection was assessed using the near infrared LI-COR fluorescent imager (LI-COR BioSciences, Lincoln, NE). A quality injection should be cleared through the bloodstream, leaving little to no near infrared signal in the tail, except for the possibility of a tiny dot at the injection site. Animals with subcutaneous injections or a ruptured vein leading to an incomplete injection were removed from the study.

### Tissue processing

Rats were euthanized at the maximum relief from hypersensitivity at 12 days post CCI (Sham ♂ = 4, ♀ = 4, CCI ♂ = 6, ♀ = 5, DF-NE ♂ = 5, ♀ = 4, CXBNE ♂ = 6, ♀ = 6) or 18 days post CCI when the animals returned to pain-like behavior (Sham ♂ = 4, ♀ = 5, CCI ♂ = 5, ♀ = 5, DF-NE ♂ = 6, ♀ = 5, CXBNE ♂ = 5, ♀ = 4) by CO_2_ asphyxiation. The sciatic nerves were immediately collected following euthanasia. All tissues were placed in 4% paraformaldehyde in 1X phosphate buffered saline (PBS) solution at 4 °C for 24 h. Tissues were then washed with 1X PBS, transferred into 30% sucrose in 1X PBS solution and kept at 4 °C until embedded. Tissues were embedded in optimal cutting temperature compound (Tissue-Tek® OCT) and frozen in a bath of isopentane. Frozen tissue blocks acclimated to the cryostat (− 25 °C) were then sectioned on the cryostat at 20 μm thickness and collected on gelatin-coated slides (SouthernBioTech, Birmingham, AL; Cat # 100241-864). Slides were kept at − 20 °C until immunofluorescence staining was performed.

### Immunofluorescence and image analysis

Tissues were stained with standard immunohistochemical protocols and recommendations of the antibody manufacturers as previously described^15,16^. Multistain procedures typically include nuclear staining (DAPI) and two other primary antibodies. Primary antibodies included an anti-CD68^53^ rabbit polyclonal antibody (ab125212) at a 1:400 dilution, an anti-CD3e mouse monoclonal antibody (MA1-90582) at a 1:500 dilution, an anti-CD4 mouse monoclonal antibody (ab33775) at a 1:500 dilution, and an anti-CD8 mouse monoclonal antibody (ab33786) at a 1:100 dilution. The secondary antibodies used were ThermoFisher’s donkey anti-rabbit Alexa Fluor 488 (A21206) and goat anti-mouse Alexa Fluor 555 (A21127), both at a 1:200 dilution. After the final washes, the slides were coverslipped with Prolong™ Diamond-DAPI (ThermoFisher).

Images were acquired and analyzed on a Nikon Eclipse Ni-U epifluorescence microscope with a 20X objective using Nikon NIS-Elements BR software (Version 5.02) for DAPI, 488 (FITC), 555 (TRITC), and DIC channels. Investigators were blinded to the treatment conditions while imaging and analyzing. Images were taken between the knots from the CCI surgery where inflammation was at its highest. Infiltrating immune cells were only counted if present within the fasciculated nerve bundle and not within the epineural sheath; this was determined by using the DIC overlay.

Quantification of macrophages and T cells used 7 regions of interest (140 μm × 140 μm) that were placed on the transmitted light image of the fasciculated axons of the sciatic nerve using the DIC view, and then individual CD68 positive or CD3e positive cells were counted. The average number of cells per region was compared across conditions.

When examining the percentage of CD68+ macrophages with the nanoemulsion, images were acquired on a Nikon Ti2 Eclipse inverted A1r confocal microscope. During this analysis, all macrophages in a field of view (212 µm X 212 µm) were counted, and then the number of those containing nanoemulsion were divided by the total number of macrophages in the field of view. Each animal was averaged within their treatment group to determine the percent of nanoemulsion-positive macrophages per sex and treatment.

Counting CD8-positive cells that infiltrated the fasciculated nerve required a different approach. Here, we counted the number of positive cells within the fasciculated nerve and divided this number by the total area counted. The cells per given area were then normalized.

### Statistical analysis

All statistical analyses were performed using GraphPad 9.0.2 Prism Software. Quantification of the gram-force measures of pain-like withdrawal behavior utilizing the von Frey up-down method used a calculation of the 50% withdrawal thresholds^28^ (50% withdrawal threshold = (10^[X^_f_^+ Kδ]^)/10,000)). The values for each time point, comparing sex and condition, were then analyzed by two-way ANOVA. Tukey’s post hoc analysis for multiple comparisons of group means was performed following two-way ANOVA. The confidence interval was 95%, and the behavioral data displayed are presented as the mean ± SEM.

The immunofluorescent imaging data comparing macrophage, CD3e+ and CD8+ cell infiltration used two-way ANOVA to compare sex and treatment ANOVAs were used in conjunction with Tukey’s post hoc analysis. For treatment, sex effects were examined using an unpaired two-tailed t-test. All immunofluorescent imaging data were calculated with a confidence interval of 95% and are displayed as the mean ± SEM with individual values plotted on the bar.

## Supplementary Information

Supplementary Information.
