# Supplementary material for: Behavioral and inflammatory sex differences revealed by celecoxib nanotherapeutic treatment of peripheral neuroinflammation
Source: Sci Rep. 2022 May 30;12:8472. doi: 10.1038/s41598-022-12248-8 (PMC9151909; doi:10.1038/s41598-022-12248-8)
Supplement: Supplementary file 1 — Supplementary Information. [file 41598_2022_12248_MOESM1_ESM.docx]

**Supplementary Figure 1.**


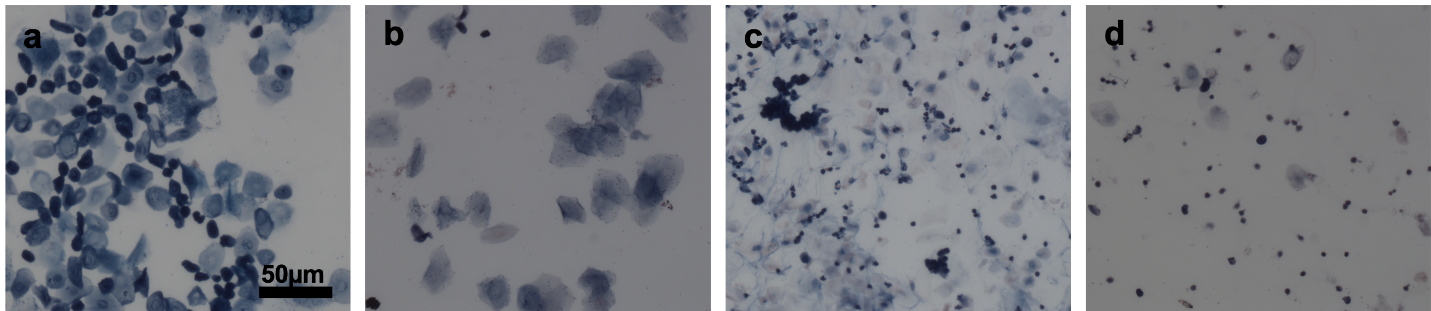


**Supplementary Figure 1.** Vaginal smears were used to stage the ~ 4-day estrous cycle of all of the female rats; with the CXB-NE females all being cycled together in estrus such as seen in panel b. **a.** Proestrus is characterized by the vast majority of cells being nucleated epithelial cells with a small presence of neutrophils. **b.** Estrus is characterized by primarily anucleated keratinized epithelial cells. **c.** Metestrus is characterized by the combination of neutrophils and anucleated keratinized epithelial cells where neutrophils begin to outnumber epithelial cells and clump together. **d.** Diestrus is characterized by low numbers of cells with small numbers of both neutrophils and epithelial cells.

**Supplementary Figure 2.**


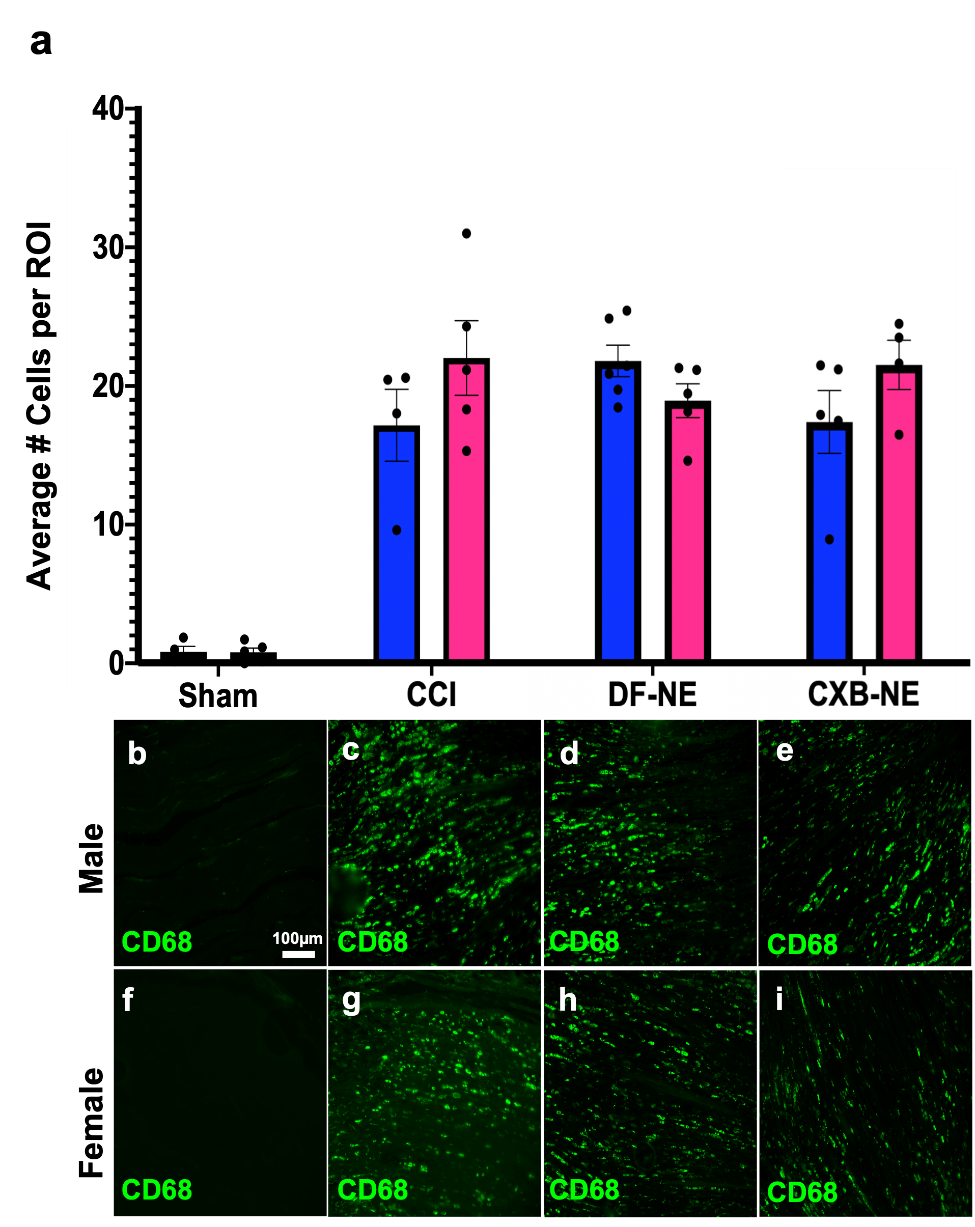


**Supplementary Figure 2.** Day 18 macrophage infiltration in CXB-NE heightens to a similar level of CCI and DF-NE as they return to hyperalgesia. **a.** Average number of CD68+ macrophages per ROI across sex and condition. Data is displayed as averages ± SEM. Sham n = 4 (male), 4 (female); CCI n = 4 (male), 4 (female); DF-NE n = 6 (male), 5 (female); and CXB-NE n = 5 (male), 4 (female). **b-i.** Immunofluorescence staining for CD68+ macrophages in the sciatic nerve.
